# Immunogenicity of rVSVΔG-ZEBOV-GP Ebola vaccination in exposed and potentially exposed persons in the Democratic Republic of the Congo

**DOI:** 10.1073/pnas.2118895119

**Published:** 2022-02-02

**Authors:** Nicole A. Hoff, Anna Bratcher, J. Daniel Kelly, Kamy Musene, Jean Paul Kompany, Michel Kabamba, Placide Mbala-Kingebeni, Bonnie Dighero-Kemp, Gregory Kocher, Elizabeth Elliott, Cavan Reilly, Megan Halbrook, Benoit Ilunga Kebela, Adva Gadoth, Guillaume Ngoie Mwamba, Merly Tambu, David R. McIlwain, Patrick Mukadi, Lisa E. Hensley, Steve Ahuka-Mundeke, George W. Rutherford, Jean Jacques Muyembe-Tamfum, Anne W. Rimoin

**Affiliations:** ^a^Department of Epidemiology, University of California, Los Angeles, CA 90095;; ^b^Department of Epidemiology and Biostatistics, University of California, San Francisco, CA 94158;; ^c^Institute for Global Health Sciences, University of California, San Francisco, CA 94158;; ^d^F.I. Proctor Foundation, University of California, San Francisco, CA 94143;; ^e^Department of Epidemiology, National Institute of Biomedical Research, Kinshasa, Democratic Republic of the Congo;; ^f^Expanded Programme for Immunization, Kinshasa, Democratic Republic of the Congo;; ^g^Integrated Research Facility, National Institute of Allergy and Infectious Diseases, National Institutes of Health, Frederick, MD 21701;; ^h^Division of Biostatistics, University of Minnesota, Minneapolis, MN 55455;; ^i^Division of Disease Control, Ministry of Health, Kinshasa, Democratic Republic of Congo;; ^j^Department of Pathology, Stanford University, Stanford, CA 94304

**Keywords:** Ebola virus disease, Ebola vaccine, rVSVΔG-ZEBOV-GP, immunogenicity, Democratic Republic of the Congo

## Abstract

Despite more than 300,000 rVSVΔG-ZEBOV-glycoprotein (GP) vaccine doses having been administered during Ebola virus disease (EVD) outbreaks in the Democratic Republic of the Congo (DRC) between 2018 and 2020, seroepidemiologic studies of vaccinated Congolese populations are lacking. This study examines the antibody response at 21 d and 6 mo postvaccination after single-dose rVSVΔG-ZEBOV-GP vaccination among EVD-exposed and potentially exposed populations in the DRC. We conducted a longitudinal cohort study of 608 rVSVΔG-ZEBOV-GP–vaccinated individuals during an EVD outbreak in North Kivu Province, DRC. Participants provided questionnaires and blood samples at three study visits (day 0, visit 1; day 21, visit 2; and month 6, visit 3). Anti-GP immunoglobulin G (IgG) antibody titers were measured in serum by the Filovirus Animal Nonclinical Group anti-Ebola virus GP IgG enzyme-linked immunosorbent assay. Antibody response was defined as an antibody titer that had increased fourfold from visit 1 to visit 2 and was above four times the lower limit of quantification at visit 2; antibody persistence was defined as a similar increase from visit 1 to visit 3. We then examined demographics for associations with follow-up antibody titers using generalized linear mixed models. A majority of the sample, 87.2%, had an antibody response at visit 2, and 95.6% demonstrated antibody persistence at visit 3. Being female and of young age was predictive of a higher antibody titer postvaccination. Antibody response and persistence after Ebola vaccination was robust in this cohort, confirming findings from outside of the DRC.

## Significance

This paper describes findings from a seroepidemiologic study involving a cohort of Ebola-vaccinated individuals from North Kivu, Democratic Republic of the Congo (DRC), who were studied as part of a collaborative effort between American and Congolese scientists and epidemiologists. Our study examines antibody response at 21 d and 6 mo postvaccination after single-dose rVSVΔG-ZEBOV-GP vaccination among Ebola virus disease–exposed and potentially exposed populations in the DRC. At 21 d of follow-up, 87.2% had an antibody response. Additionally, 95.6% demonstrated antibody persistence at 6 mo of follow-up. These findings give crucial evidence that antibody response and persistence after Ebola vaccination is robust in outbreak settings in the DRC, knowledge that significantly informs the use of vaccination for outbreak control.

Since the rVSVΔG-ZEBOV-glycoprotein (GP) vaccine completed clinical trials in West Africa, over 300,000 doses of the vaccine have been deployed in response to the multiple Ebola virus disease (EVD) outbreaks in the Democratic Republic of the Congo (DRC). While initially deployed under a “compassionate use/expanded access” protocol (1, 2), as of December 19, 2019, the vaccine was officially licensed by both the American (Food and Drug Administration, FDA) and European (European Medicines Agency) regulatory agencies (3, 4). Wide use of this vaccine was supported by evidence gathered in clinical trials and other studies, including those postlicensure conducted in North America and West Africa, which demonstrated short-term vaccine efficacy (5–16). In addition to short-term protection, clinical trials and other studies have provided evidence of Ebolavirus Zaire (EBOV)–specific antibody persistence up to 2 y postvaccination, suggesting that the vaccine may continue to offer protective immunity over time (5, 7, 8, 14, 15). While promising, observations of successful rVSVΔG-ZEBOV-GP vaccine performance in outbreak settings have mostly come from studies conducted at the end of the 2014 to 2016 West African EVD outbreak (7, 13, 14, 17). Such studies in the DRC are lacking.

Furthermore, recent evidence of breakthrough infections within the DRC has highlighted the need for DRC-specific vaccine research, including magnitude and durability of serological response after rVSVΔG-ZEBOV-GP vaccination in Congolese populations. In April 2019, the World Health Organization (WHO) released a preliminary report of rVSVΔG-ZEBOV-GP efficacy in the 2018 to 2020 Beni outbreak. Among 93,965 people at risk who were vaccinated, there were 15 confirmed EVD cases with onset of symptoms 10 d or more postvaccination (18). Another report describes an individual who presented with EVD 6 mo after vaccination, initiating a chain of transmission resulting in 91 subsequent infections (19), prompting questions around the duration of protection. These recent events highlight both the consequences of breakthrough infections and the possibility of waning immunity postvaccination.

When considering rVSVΔG-ZEBOV-GP performance in the DRC, there are several factors that may impact the effect of vaccination in Congolese populations. First, an increase in vaccination dose could have resulted in increased immunogenicity in the DRC. Vaccination deployment during the EVD outbreak of 2018 initially included double the plaque-forming units (PFUs) in the vaccine dosage compared to what was used in West Africa (20 million PFU/mL versus 10 million PFU/mL, respectively) (20). As previous studies have identified varying immunogenicity after different vaccine doses in different locations, this variation in vaccine dose could lead to differing antibody responses from previously studied cohorts (15). Second, an important component of the vaccine deployment was the requirement for an ultracold chain (storage of vaccine at −70 °C), which poses extreme logistical challenges in resource-constrained environments. Despite considerable efforts to avoid cold chain failures, it is plausible that fluctuations could have occurred and caused changes in vaccine effectiveness (21). Third, populations in this region may have a baseline level of filovirus seroreactivity that may enable a more robust response to Ebola vaccination (15, 22). Previous serologic studies in the DRC have indicated that Congolese populations may not be naive to filovirus exposures, with individuals presenting evidence of robust antibody responses to various filoviruses in the absence of a known history of EVD (23–26). While there had never been a reported EVD outbreak in North Kivu prior to 2018, this province is known for highly mobile populations; proximity to large forested areas, which may harbor filovirus or filovirus-like pathogens; and access to cross-border populations, including those from Uganda, which have had previous filovirus outbreaks (27–29). Finally, the underlying prevalence of immunosuppressive conditions, such as HIV infection and poor nutritional status, could hinder vaccine immunogenicity in Congolese populations (30).

Given the DRC’s unique landscape, which includes evidence of breakthrough infections, a more thorough region-specific understanding of serologic response to Ebola vaccination is needed. Various factors such as vaccine dose, storage conditions, current infections, and previous exposure may alter the magnitude and durability of antibody response after vaccination in Congolese populations (7, 31, 32). To better understand rVSVΔG-ZEBOV-GP performance in the DRC, we conducted a seroepidemiologic study of postvaccination antibody persistence in Congolese populations, who may have meaningfully different experiences than those in West Africa. Here, we provide a preliminary report of antibody response and persistence, along with potential predictors, after single-dose rVSVΔG-ZEBOV-GP vaccination among EVD-exposed and potentially exposed populations in the DRC.

## Results

The North Kivu cohort includes 608 eligible vaccinated individuals without elevated baseline antibody titers (Table 1). At enrollment, average time elapsed between vaccination and first study visit was 0.1 d (SD = 0.6). Most participants were male (64%), married (57%), and between the ages of 20 and 39 (58%). Our sample population reported various levels of highest education, with 41% having any primary school or apprenticeship, 26% having finished secondary school, and 29% having college, university, or graduate education. The small remainder of participants (4%) reported having no formal education. Additionally, 41% of participants reported being a health care worker. Thirty-two percent of the sample reported having contact with a confirmed, probable, or suspected EVD case, while 66% reported not having such contact. Two percent of the sample did not know their EVD contact history.

**Table 1.** Sample characteristics of 608 rVSVΔG-ZEBOV-GP vaccine recipients from Beni and the surrounding areas in the DRC, August 2018

	No. of participants	%
Mean age, y*	35.4	13.2
Mean time since vaccination, d*	0.1	0.6
Sex		
Male	388	63.8
Female	220	36.2
Age, y		
12–19	60	9.9
20–29	156	25.7
30–39	198	32.6
40–49	112	18.4
50–82	82	13.5
Education^†^		
None	22	3.6
Any primary school or apprenticeship	248	40.9
Finished secondary school	160	26.4
College/university or graduate school	177	29.2
Marital status^‡^		
Single	243	40.2
Married or living together as married	346	57.3
Divorced, separated, or widowed	15	2.5
Currently a health care worker?^‡^		
Yes	245	40.6
No	359	59.4
Has ever had contact with a confirmed, probable, or suspected EVD case?^§^		
Yes	176	32.2
No	359	65.6
Don’t know	12	2.2
Days since vaccination^¶^		
1	552	91.9
2	29	4.8
3	19	3.2
4	1	0.2

*Data are presented as mean (SD).

^†^One missing response.

^‡^Four missing responses.

^§^Sixty-one missing responses.

^¶^Seven missing responses.

**Fig. 1.**
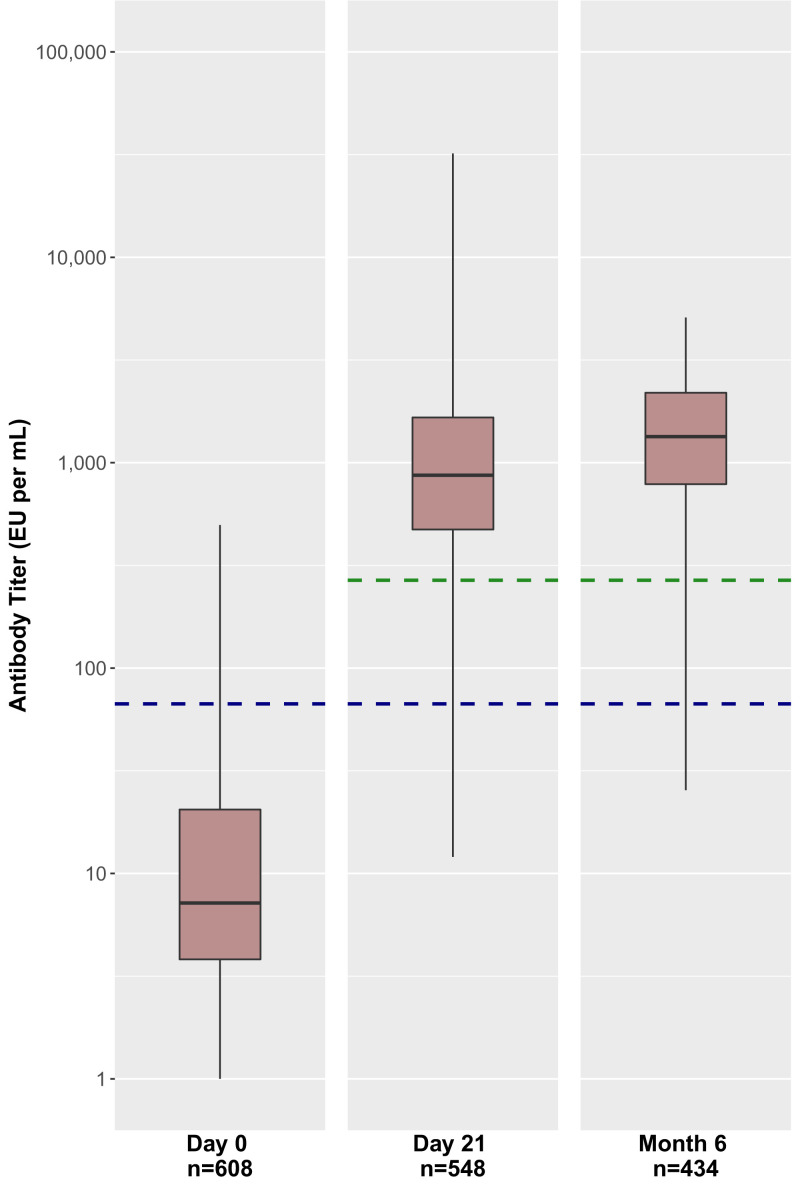
Antibody titers following rVSVΔG-ZEBOV-GP vaccination among 608 participants in Beni, DRC. The blue dashed line indicates 66.96 EU/mL, the LLOQ for FANG ELISA. The green dashed line indicates 267.85 EU/mL, four times the LLOQ for FANG ELISA, or the lower limit for which our participants could be considered positive for antibody response or persistence. Box plots show the median, interquartile range, and range of antibody titers at each time point for individuals who did not have an elevated titer at baseline.

At baseline (visit 1), participants had a geometric mean antibody titer measured at 9 EU/mL (95% CI, 8, 10) (Table 2), which was below the lower limit of quantification (LLOQ) of 66.96 EU/mL for the Filovirus Animal Nonclinical Group anti-Ebola virus glycoprotein immunoglobulin G enzyme-linked immunosorbent assay (FANG ELISA). Retention in the sample varied by follow-up, in which 548 participants (90.1%) completed a 21-d follow-up visit and 434 (71.4%) completed a 6-mo follow-up visit. Of the 548 returning participants, 478 (87.2%) had an antibody response at 21 d postvaccination (visit 2), and of the 434 returning at 6 mo, 415 (95.6%) demonstrated antibody persistence (visit 3). Of note, at the 21-d time point, 9 participants had titers higher than 10,000 EU/mL. In contrast, no participants showed titers higher than 10,000 EU/mL at the 6-mo time point (Fig. 1).

**Table 2.** Antibody response and persistence during follow-up among 608 rVSVΔG-ZEBOV-GP vaccine recipients without elevated baseline titers

	rVSVΔG-ZEBOV-GP recipients
At baseline	
No. of participants	608
Geometric mean titer (95% CI), EU/mL	9 (8, 10)*
At 21 d	
No. of participants	548
Geometric mean titer (95% CI), EU/mL	900 (826, 981)
Participants with antibody response (95% CI), %	87.2 (84.1, 89.9)
At 6 mo	
No. of participants	434
Geometric mean titer (95% CI), EU/mL	1,231 (1,145, 1,324)
Participants with antibody persistence (95% CI), %	95.6 (93.3, 97.3)

*Below the LLOQ of 66.96 EU/mL.

There was some evidence that demographic factors may have been associated with vaccine response (Tables 3 and 4). In both univariate and multivariable analyses, female participants had significantly higher antibody titers than male participants at 6-mo follow-up. Additionally, age seemed to show an association with antibody response and duration in univariate and multivariable models. Overall, individuals above 19 y of age tended to show lower antibody titers at 21-d and 6-mo postvaccination compared to those between 12 and 19 y of age. In the multivariable model, the reduction in antibody response was significant for those between 30 and 49 y of age. Meanwhile, the reduction in antibody persistence was significant for only those between 20 and 39 y old in the multivariable model. In univariate models only, being a health care worker was associated with a poorer antibody response at 21 d, while being single was associated with a higher titer at the same time point. Given that these univariate relationships no longer hold significance in the multivariable model, it is possible that these effects are the product of confounding by age. Ultimately, despite differences, none of the subgroups had median titer levels below 607 EU/mL at either the 21-d or 6-mo time periods.

**Table 3.** Univariate analysis for predictors of antibody response at 21 d and 6 mo of follow-up for rVSVΔG-ZEBOV-GP vaccine recipients in Beni, DRC

	21 d (*n* = 548)		6 mo (*n* = 434)	
	Antibody titer ratio	95% CI	Antibody titer ratio	95% CI
Sex				
Male	Reference		Reference	
Female	1.11	0.93, 1.32	1.35	1.15, 1.58
Age, y				
12–19	Reference		Reference	
20–29	0.72	0.54, 0.96	0.67	0.51, 0.90
30–39	0.65	0.49, 0.87	0.65	0.49, 0.86
40–49	0.56	0.41, 0.75	0.75	0.56, 1.01
50–82	0.66	0.47, 0.93	0.84	0.61, 1.17
Education*				
None	0.66	0.43, 1.03	0.77	0.52, 1.14
Any primary school or apprenticeship	Reference		Reference	
Finished secondary school	1.05	0.83, 1.31	0.89	0.74, 1.07
College/university or graduate school	0.87	0.71, 1.06	0.96	0.81, 1.14
Marital status^†^				
Single	1.22	1.03, 1.46	1.07	0.93, 1.25
Married or living together as married	Reference		Reference	
Divorced, separated, or widowed	1.13	0.63, 2.05	1.25	0.77, 2.03
Currently a health care worker?^†^				
Yes	0.83	0.70, 0.99	0.98	0.85, 1.14
No	Reference		Reference	
Has ever had contact with a confirmed, probable, or suspected EVD case?^‡^				
Yes	0.87	0.72, 1.05	0.96	0.81, 1.14
No	Reference		Reference	
Don’t know	0.91	0.66, 1.27	1.05	0.81, 1.36

*One missing response.

^†^Four missing responses.

^‡^61 missing responses.

**Table 4.** Multivariable analysis for predictors of antibody response at 21 d and 6 mo of follow-up for 539 rVSVΔG-ZEBOV-GP vaccine recipients in Beni, DRC

	21 d		6 mo	
	Antibody titer ratio	95% CI	Antibody titer ratio	95% CI
Sex				
Male	Reference		Reference	
Female	1.11	0.92, 1.33	1.36	1.15, 1.62
Age				
12–19	Reference		Reference	
20–29	0.73	0.53, 1.01	0.69	0.49, 0.97
30–39	0.68	0.48, 0.97	0.62	0.44, 0.89
40–49	0.59	0.40, 0.86	0.71	0.48, 1.05
50–82	0.71	0.47, 1.07	0.80	0.54, 1.19
Education				
None	0.72	0.46, 1.12	0.80	0.53, 1.21
Any primary school or apprenticeship	Reference		Reference	
Finished secondary school	1.15	0.90, 1.46	0.99	0.81, 1.21
College/university or graduate school	1.00	0.80, 1.25	1.10	0.91, 1.34
Marital status				
Single	1.00	0.79, 1.25	0.94	0.77, 1.14
Married or living together as married	Reference		Reference	
Divorced, separated, or widowed	1.18	0.67, 2.10	1.03	0.63, 1.70
Currently a health care worker?				
Yes	0.90	0.74, 1.09	1.06	0.90, 1.26
No	Reference		Reference	
Has ever had contact with a confirmed, probable, or suspected EVD case?				
Yes	0.91	0.74, 1.12	0.94	0.79, 1.13
No	Reference		Reference	
Don’t know	1.03	0.68, 1.55	1.08	0.77, 1.51

## Discussion

The results of this study indicate that antibody response and persistence postvaccination with the Merck(R) rVSVΔG-ZEBOV-GP vaccine was robust, aligning with existing literature from outside of the DRC (5–16, 33). We observed an increase at each time point in the geometric mean, potentially indicating that participants were having increased antibody response as time went on. This observation is most likely a result of vaccine response but also potentially the product of natural exposure in our cohort, as the outbreak was ongoing during the study period. Our study sample, which received vaccination containing 20 million PFUs/mL, showed similar response levels compared to other cohorts of adults in low- and middle-income countries who received intermediate or high doses of this vaccine (3 million and 20 million PFUs, respectively) (15). While a small number of participants in this study did not mount an antibody response, rVSVΔG-ZEBOV-GP vaccination appears to generally lead to a robust antibody response. However, vaccine efficacy could not be calculated with this study. Further research should seek to determine the correlation between anti-GP antibody response and EBOV protection so that vaccine efficacy can be estimated through measures of immunogenicity.

In this cohort, female participants were more likely to have a higher antibody response than male participants at the 6-mo follow-up visit. While the PREVAIL I study did not detect differences by sex in antibody response for rVSVΔG-ZEBOV-GP (16), previous research on other vaccines has noted that female persons typically have a higher antibody response than male persons (34–36). Data from a study on the smallpox vaccine, Dryvax, showed that female persons have increased humoral immunity and B-cell responses postvaccination (37). It is hypothesized that female persons may be more resistant to infectious diseases and mount more robust vaccine responses because of more active immune systems compared to male persons, the same biological mechanism that renders them at higher risk for autoimmune disorders (37–39). Our data on responses to rVSV-ZEBOV appear to corroborate this hypothesis and add to that growing body of evidence (37–39). Additionally, it is possible that this observation is a result of gender differences in risk behaviors during the follow-up period. For example, if female persons were more likely perform funeral rites or be health care workers with direct patient contact such as nurses, they may have had continuous exposure throughout the follow-up period. If so, this continuous exposure could have contributed to higher titers in this group.

We also observed significant differences in vaccine antibody response across different age categories. As expected, the youngest participants had the highest antibody response both 21 d and 6 mo postvaccination. Adolescents (12- to 19-y-olds) may have more robust immune systems and have higher ability to produce a robust immune response postvaccination with a progressive decline in immune system function with age (40, 41). Though not all age categories significantly differed from the reference group (12- to 19-y-olds), point estimates showed a general pattern of having a decreased antibody titer within older age groups at both time points. It is possible that this lack of significance across all categories may have been due to sparse data after separation by age group.

There were a number of limitations to this study. Our study enrolled a convenience sample of limited sample size without a defined control group because of resource constraints and safety concerns associated with data collection in an active EVD outbreak in an area with armed conflict. If those who were enrolled out of convenience differed meaningfully from those who were not enrolled, the generalizability of our findings may be limited because of our sampling scheme. Additionally, in efforts not to disrupt outbreak response, we were unable to conduct enrollment procedures prior to vaccination and enrolled participants after the 30-min observation period postvaccination. However, it is unlikely that individuals would mount or show an antibody response in the hours postvaccination. Retention at 21 d and 6 mo postvaccination was just over 70%, partially because of a highly mobile population and continuing armed conflict. Additionally, it is possible that some participants could have died of EVD prior to follow-up visits. If loss to follow-up was associated with both predictors and antibody titers measures at follow-up visits, our estimates in Tables 3 and 4 could be subject to selection bias. During laboratory testing, we employed a two-operator duplicate procedure with multiple quality checks for each plate to ensure low intra- and interplate variability. Samples that failed quality controls were retested and verified by an external laboratory (National Institute of Allergy and Infectious Diseases Integrated Research Facility, Fort Detrick, MD). Lastly, correlates of protection have not been fully described for EVD, and therefore, our discussion of antibody titers cannot be used to draw conclusions regarding the protection conferred by vaccination.

Overall, our results indicate a robust antibody response after vaccination with the rVSVΔG-ZEBOV-GP vaccine, which persists 6 mo postvaccination. However, not everyone in our sample showed an antibody response postvaccination; 70 individuals (12.8%) at 21 d postvaccination and 19 individuals (4.4%) 6 mo postvaccination failed to meet our definition of antibody response and persistence. Despite these nonresponders and broader evidence of breakthrough cases in the DRC (18, 19), our study suggests that most Congolese individuals exhibit a strong antibody response postvaccination. Future research must determine the correlation between serological vaccine response and conferred protection, along with the duration of each among individuals in the DRC.

## Methods

### Study Design, Setting, and Participants.

We conducted a longitudinal cohort study of consenting individuals who received the rVSVΔG-ZEBOV-GP vaccine during the North Kivu EVD outbreak in North Kivu Province, DRC. Between August and September 2018, we worked alongside the Expanded Program for Immunization (EPI) of the Ministry of Health and WHO to obtain a convenience sample of eligible participants. During our recruitment period, the rVSVΔG-ZEBOV-GP vaccine was not yet licensed and was administered as part of a “compassionate use/expanded access” protocol using ring vaccination (1, 42). As a result, individuals were eligible for the vaccine and, by default, enrollment in this study if they were included in the following groups: 1) contacts or “contacts of contacts” of confirmed EVD cases (ring vaccination) or 2) health care/frontline workers in EVD-affected or potentially affected areas. At the time of enrollment, children under 1 y of age, pregnant women, and lactating women were excluded from vaccination and thus not eligible for this study. These restrictions were eventually removed for vaccination in 2019.

### Study Procedures.

Study visits occurred at the following three time points: 1) day 0, at least 30 min postvaccination, after individuals had been monitored for potential adverse events; 2) between day 21 and 28 postvaccination, during follow-up with the EPI/WHO teams for adverse events monitoring; and 3) 6 mo postvaccination. At each study visit, consenting/assenting individuals (7 y and older) completed a structured questionnaire, underwent a basic physical assessment, and were asked to provide a blood specimen. Blood samples were collected by trained phlebotomists using venipuncture methods. Whole blood samples were collected in red top tubes (BD) for serum isolation. The electronic questionnaire using Open Data Kit. Collect application was administered by trained interviewers in the participant’s preferred local language (French or Swahili). Interview questions included demographics; eligibility group; timing since vaccination; and potential exposures to EBOV from community, health care, and animal interactions.

To ensure official vaccination activities suffered no disruptions, participants were not enrolled until after vaccination and the 30-min follow-up period and did not receive compensation at study enrollment (time of vaccination). Participants were compensated for transportation costs to and from the study site for all follow-up visits. Blood samples were processed in the field. After centrifuging blood samples for 10 min to separate the serum, serum aliquots were heat-inactivated following standard procedures at 56 °C for 30 min and then frozen at −20 °C and shipped to the Institut National de Recherche Biomedicale in Kinshasa, DRC. Samples were then stored at −80 °C before serological testing onsite in Kinshasa.

### Serologic Measurements.

Serologic testing was completed using the FANG ELISA to measure IgG antibody levels against the EBOV surface GP (anti-GP) in serum. The FANG ELISA is a quantitative immunoassay developed by Battelle Memorial Institute for the US Department of Defense Joint Program Executive Office for Chemical, Biological, Radiological, and Nuclear Defense’s Medical Countermeasures Systems Joint Vaccine Acquisition Program (43). More details can be found elsewhere (43). Anti-GP IgG antibody titers were recorded at 0 d, 21 d, and 6 mo postvaccination.

In the absence of a regional baseline value, our study defined having an elevated baseline titer as having above an antibody titer value above 607 EIA (EU)/mL, a previously used value derived from a cohort in Mali unexposed to EVD (16). Nine individuals had a titer above this limit and were excluded from the analysis. There was no lower limit to being included in the analysis, although a large majority of participants (91%) had baseline titers that were below the LLOQ of 66.96 EU/mL for FANG ELISA.

Of the remaining participants without an elevated baseline titer, antibody responses of participants were considered positive if their 21-d (visit 2) titer increased by a factor of 4 (if log_10_ titer increased by 0.6) or more from their baseline value and were at least four times the LLOQ (267.84 EU/mL). Antibody persistence was also defined as a fourfold increase in antibody titer between study visits 1 and 3 along with a visit 3 titer a least four times the LLOQ. For purposes of calculating fold changes, geometric mean, and log of antibody titer, antibody titers of 0 were artificially changed to 1 so that estimates could be calculated.

### Statistical Analyses.

We calculated the geometric mean concentrations and 95% CIs of antibody titers present at each visit and assessed each participant for an antibody response and persistence. Baseline demographics for predictors of antibody titer at follow-up were summarized with percentages of means and SDs. We assessed these relationships with univariate linear mixed models that included repeated measures of log_10_ antibody titer per participant as the response and age, sex, education, marital status, health care worker status, and EVD exposure history as predictors. A multivariable model was then built to include all the listed predictors. These models treated participants as an additive random effect with an unstructured covariance matrix and the predictors of interest as fixed effects. Once estimates for mean difference of log_10_ antibody titer and corresponding confidence intervals were obtained, these estimates were transformed out of the log scale producing antibody titer ratio estimates on the original antibody titer scale.

All analyses were conducted in SAS (version 9.4, Cary, NC) and R (version 3.2.3, R Foundation for Statistical Computing, Vienna, Austria).

### Ethics Statement.

This study was approved by Institutional Review Boards (IRBs) at the University of Kinshasa in Kinshasa, DRC (ESP/CE/022/2017) and at the University of California, Los Angeles (IRB no. 16–001346). Additionally, the study was approved by the Scientific Committee for Ebola Research during an outbreak at the National Institute of Biomedical Research under the Ministry of Health. Before any study-related procedures were conducted, participants signed or marked the approved informed consent form, and parents or guardians provided this consent on behalf of all child participants, while adolescents aged 7 to 17 provided assent as appropriate.

## Data Availability

All data used in this analysis (a subset of the study data) and a corresponding codebook are provided as supplemental files.
